# Bioinformatic Prediction of an tRNA^Sec^ Gene Nested inside an Elongation Factor SelB Gene in Alphaproteobacteria

**DOI:** 10.3390/ijms22094605

**Published:** 2021-04-27

**Authors:** Takahito Mukai

**Affiliations:** Department of Life Science, College of Science, Rikkyo University, 3-34-1 Nishi-Ikebukuro, Toshima-ku, Tokyo 171-8501, Japan; takahito.mukai@rikkyo.ac.jp

**Keywords:** genetic code, selenocysteine, non-coding RNA

## Abstract

In bacteria, selenocysteine (Sec) is incorporated into proteins via the recoding of a particular codon, the UGA stop codon in most cases. Sec-tRNA^Sec^ is delivered to the ribosome by the Sec-dedicated elongation factor SelB that also recognizes a Sec-insertion sequence element following the codon on the mRNA. Since the excess of SelB may lead to sequestration of Sec-tRNA^Sec^ under selenium deficiency or oxidative stress, the expression levels of SelB and tRNA^Sec^ should be regulated. In this bioinformatic study, I analyzed the Rhizobiales SelB species because they were annotated to have a non-canonical C-terminal extension. I found that the open reading frame (ORF) of diverse Alphaproteobacteria *selB* genes includes an entire tRNA^Sec^ sequence (*selC*) and overlaps with the start codon of the downstream ORF. A remnant tRNA^Sec^ sequence was found in the *Sinorhizobium meliloti* *selB* genes whose products have a shorter C-terminal extension. Similar overlapping traits were found in Gammaproteobacteria and Nitrospirae. I hypothesized that once the tRNA^Sec^ moiety is folded and processed, the expression of the full-length SelB may be repressed. This is the first report on a nested tRNA gene inside a protein ORF in bacteria.

## 1. Introduction

**Figure 1:**
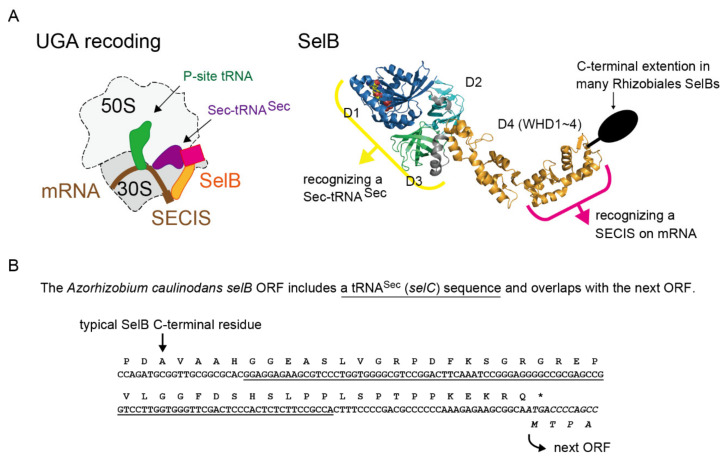
Elongation factor SelB and Sec-tRNA^Sec^. (**A**) The mechanism of the SECIS-dependent recoding of the UGA codon for Sec by the SelB●SECIS●Sec-tRNA^Sec^ complex (modified from [3,4]). Many of the Rhizobiales SelB sequences were annotated to have a C-terminal extension, which is indicated as an additional domain appended to the C-terminus of the crystal structure picture of *Aquifex aeolicus* SelB composed of four domains (PDB id: 4ZU9). (**B**) The *Azorhizobium caulinodans selC* (tRNA^Sec^) sequence is nested inside the ORF of the *selB* gene. The star indicates a translational stop signal.

Selenocysteine is the 21st amino acid used in diverse bacteria, archaea, and eukaryotes for expressing selenoproteins [1]. In this work, I focus on the bacterial system. Unlike most of the canonical amino acids, Sec is synthesized on tRNA^Sec^ molecules and delivered to a growing polypeptide in the ribosome by the dedicated elongation factor SelB. First, tRNA^Sec^ is charged with serine by seryl-tRNA synthetase. Ser-tRNA^Sec^ is then converted to Sec-tRNA^Sec^ by selenocysteine synthase (SelA) using selenophosphate synthesized by SelD [2]. SelB binds to a Sec-tRNA^Sec^ and a Sec-insertion sequence (SECIS) element on mRNA to mediate Sec-insertion (see Figure 1A) [3]. The SelB domains I/II/III are mainly responsible for the GTPase activity, while the domain IV composed of four winged helix domains (WHDs) is responsible for the SECIS recognition (Figure 1A) [3,4]. In most of the Sec-utilizing bacteria, the UGA stop codon directly followed by a SECIS element is recoded in a competition with release factor 2 [5], while some bacteria use the UAG stop codon or the UGC/UGU cysteine codons together with anticodon variants of tRNA^Sec^ [6].

The primary role of Sec in common bacteria such as Alphaproteobacteria, Betaproteobacteria, and Gammaproteobacteria species is to express Sec-containing formate dehydrogenases (FDHs) [7]. Formate metabolism is important for nitrogen fixation and formate-dependent respiration in Rhizobiales [8,9]. It is known that a megaplasmid pSymA of *Sinorhizobium* (*Rhizobium*) *meliloti* encode a *fdoGHI* (for FDH-O) and *selABCD* gene cluster [10,11]. In our previous study on non-canonical SelB sequences [12], it was found that Rhizobiales SelB sequences in the public databases are annotated to have a C-terminal extension compared to other bacterial SelB sequences (Figure 1A) [13]. In the present study, a careful analysis of these Rhizobiales SelB sequences was performed to reveal the possible reason.

## 2. Results

### 2.1. tRNA^Sec^ is Encoded Inside the selB Gene in Diverse Alphaproteobacteria

**Figure 2:**
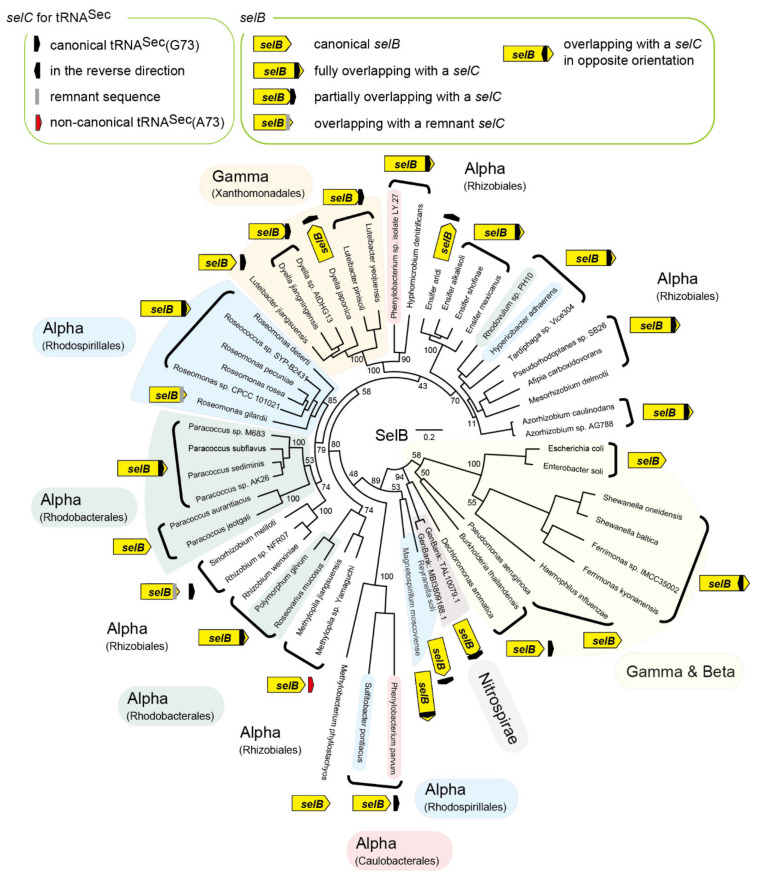
SelB phylogenetic tree showing the distribution of *selB-selC* overlapping traits in diverse bacteria. Alphaproteobacteria, Betaproteobacteria, Gammaproteobacteria, and Nitrospirae were mainly investigated in this study. This unrooted tree was developed by maximum likelihood estimation using the JTT matrix-based model with 100 replicates. The bootstrap values (percentages) are shown on the tree.

**Figure 3:**
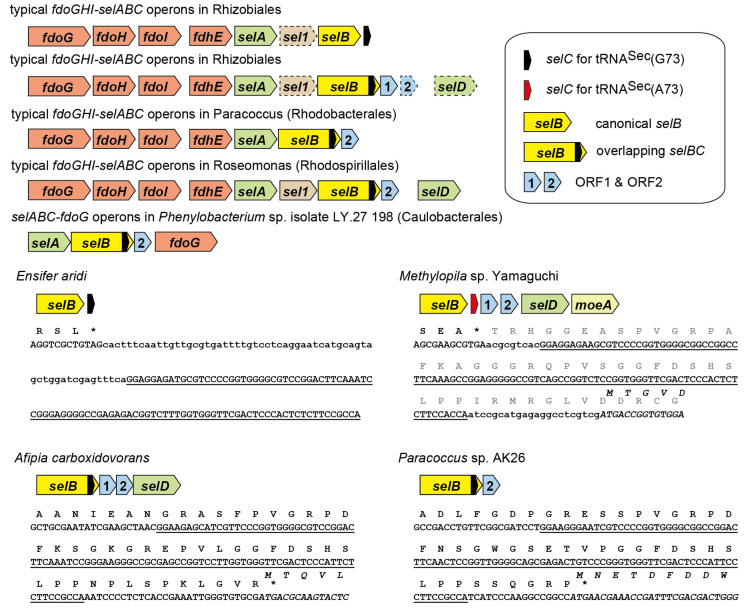
Bioinformatic analysis of *fdoGHI* and *selABC* gene clusters in four orders of Alphaproteobacteria (Rhizobiales, Rhodobacterales, Rhodospirillales, Caulobacterales). The *fdoGHI* genes encode a membrane-bound formate dehydrogenase (FDH-O) carrying a catalytic Sec residue. The SelD proteins synthesize selenophosphate which is the selenium donor used by SelA. The SelD and Sel1-like proteins are not selenoproteins in these bacteria. The two ORFs associating with the overlapping *selBC* gene were named ORF1 and ORF2 in this study. As reported previously [12], *Methylophila* spp. use a tRNA^Sec^ with a non-canonical discriminator base A73 instead of G73. Sequence information of the overlapping *selBC* genes were provided. The stars indicate a translational stop signal. The tRNA^Sec^ sequences were underlined. The reading frames of ORF1 or ORF2 were italicized.

As shown in Figure 1B, it was found that an entire tRNA^Sec^ sequence is nested inside the ORF of the *selB* gene of an Rhizobiales bacterium *Azorhizobium caulinodans*. The stop codon of the *selB* gene overlaps with the start codon of the next ORF. Thus, the C-terminal extension results from the translation of the tRNA^Sec^ sequence into amino acids. Since overlapping of stop and start codons is common for polycistronic mRNAs and may support translation re-initiation [14], the annotation of the ORFs may be true. Is this common in Rhizobiales and in other orders of Alphaproteobacteria? I performed a bioinformatic analysis of *selB* and *selC* genes. In Figure 2, the distribution of the *selB-selC* overlapping trait is overlaid on the phylogenetic tree of SelB sequences analyzed in this study. In summary, I found several types of overlapping genes and the penetrance of the overlapping trait even outside the Alphaproteobacteria class. In Alphaproteobacteria, diverse species of Rhizobiales and a few groups of Rhodobacterales, Rhodospirillales, and Caulobacterales have a *selC* nested inside the *selB* gene (Figure 3). In many cases, the stop codon of the *selB* ORF overlaps with the start codon of the next ORF. In most cases, the *selC*-encoded tRNA^Sec^ sequence is 96 nucleotide residues in length or 93 residues (due to the lack of the CCA tail in the *selC* gene), which may facilitate the maintenance of the reading frame. Some species have lost the overlap (Figure 3); *Methylopila* species have a new stop codon before the *selC* sequence, while some *Ensifer* species have a long spacer between the *selB* and *selC* genes. These results clearly suggested that the overlapping trait is highly conserved but is not essential.

### 2.2. tRNA^Sec^ Remnant is Encoded inside the selB Gene in pSymA

**Figure 4:**
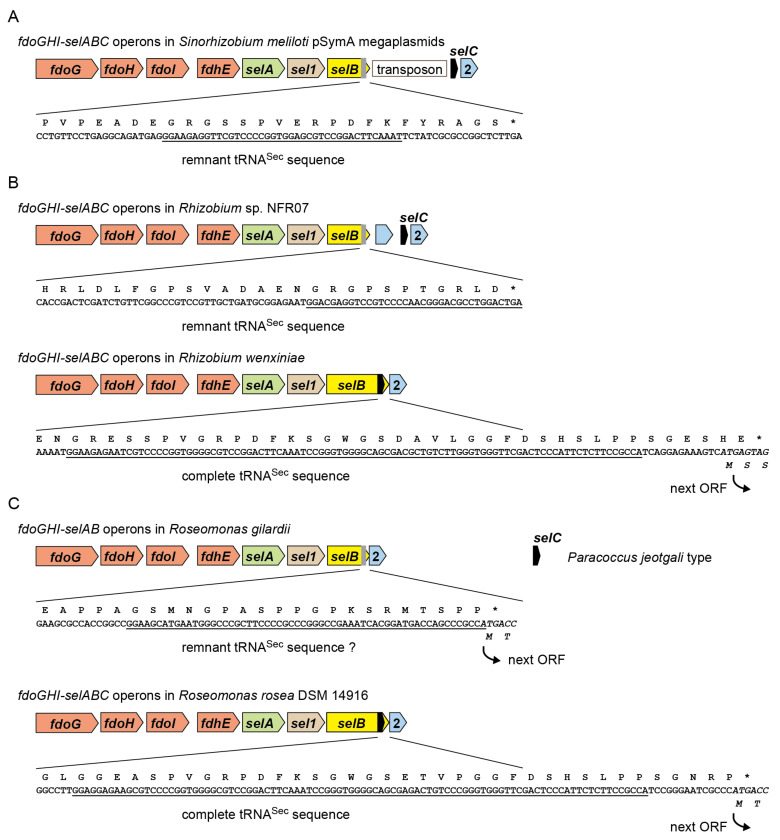
Remnant tRNA^Sec^ sequence in the *selB* gene. The stars indicate a translational stop signal. The tRNA^Sec^ sequences were underlined. The reading frames of ORF2 were italicized. (**A**) In the megaplasmid pSymA of *Sinorhizobium meliloti*, the original tRNA^Sec^ sequence in the overlapping *selBC* gene had been disrupted by the insertion of a transposon. (**B**) In a Rhizobium lineage, the original tRNA^Sec^ sequence in the overlapping *selBC* gene was disrupted by the insertion of a small protein gene, while the overlapping *selBC* gene arrangement remains in another lineage. (**C**) In *Roseomonas gilardii* and *R. mucosa*, the *selB* gene encodes a short C-terminal extension and overlaps with the ORF2 gene, although no clear trace of tRNA^Sec^ was found in the *selB* gene. A *selC* gene resembling that of *Paracoccus jeotgali* strain CBA4604 exists elsewhere in their genome. On the other hand, the overlapping *selBC* gene remains in other *Roseomonas* lineages. This suggested that the SelB C-terminal extension and the translational coupling of the *selB* and ORF2 genes may have some importance.

It is known that the *selAB* genes and the *selCD* genes are separated by a transposon in *S. meliloti* pSymA megaplasmid [10,11]. However, the *S. meliloti* SelB has a C-terminal extension [13] which is slightly shorter than that of *A. caulinodans* SelB. It was revealed that the 5′ half of the overlapping tRNA^Sec^ sequence remains in the *S. meliloti selB* ORF (Figure 4A). Thus, the shorter C-terminal extension results from the translation of the remnant tRNA^Sec^ sequence into amino acids. The transposon insertion may have generated a new stop codon for the pSymA *selB* gene. A similar case was found in a lineage of *Rhizobium* (Figure 4B). *Rhizobium* sp. NFR07 has a remnant tRNA^Sec^ sequence in the *selB* gene, while *Rhizobium wenxiniae* has a complete tRNA^Sec^ sequence nested in the *selB* gene. *Roseomonas gilardii* and *R. mucosa* also have a SelB with a shorter C-terminal extension, while their remnant tRNA^Sec^ sequences seem to be highly degenerate (Figure 4C). The C-terminal extension may facilitate the translational coupling of the *selB* ORF and the ORF2 in these *Roseomonas* lineages (Figure 4C).

### 2.3. tRNA^Sec^ Partially Overlapping with the selB Gene in Gammaproteobacteria

**Figure 5:**
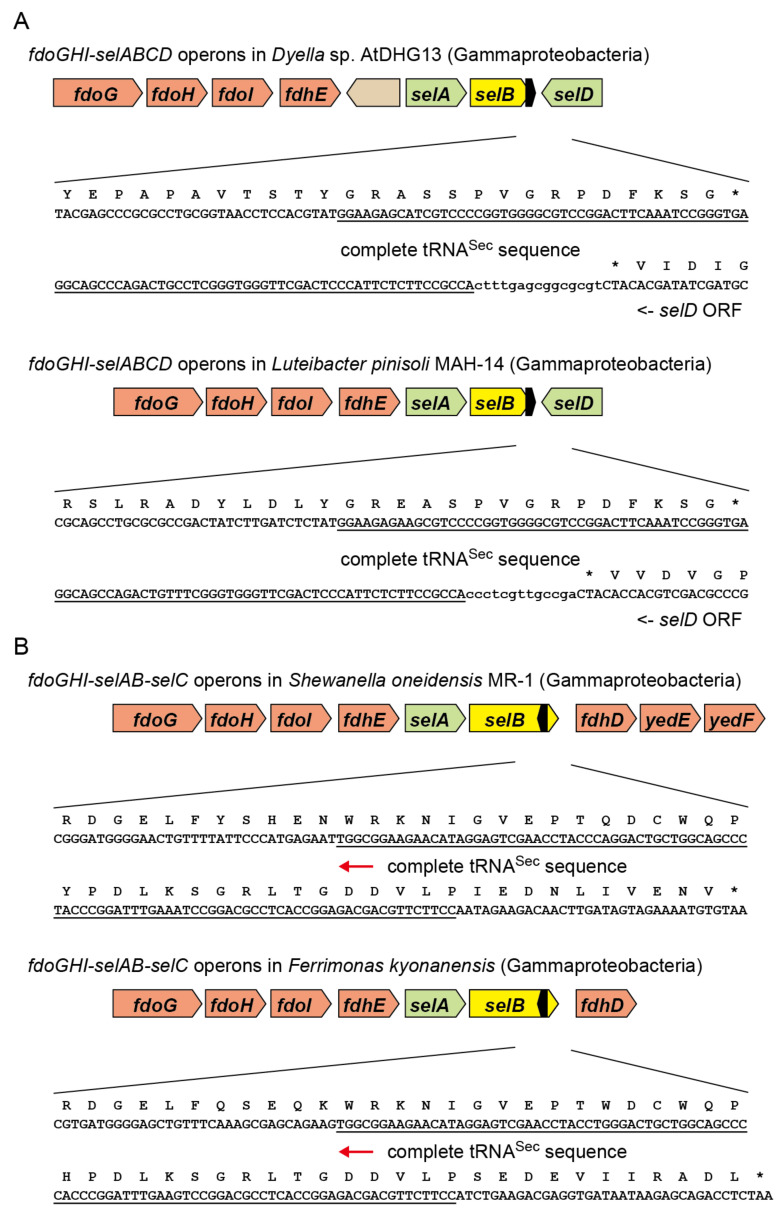
Partially overlapping *selB*-*selC* genes in Gammaproteobacteria. The stars indicate a translational stop signal. The tRNA^Sec^ sequences were underlined. (**A**) A few lineages of *Dyella* and *Luteibacter* in Gammaproteobacteria have a partially-overlapping *selB* and *selC* genes. The stop codons for the *selB* genes were found in the same position of the *selC* genes. In other *Dyella* and *Luteibacter* species, the *selB* stop codons precede the *selC* sequence. This may imply that the two *Dyella* and *Luteibacter* lineages have inherited the original *selB-selC* genes partially overlapping each other, which may have been derived from Alphaproteobacteria through horizontal gene transfer. (**B**) *Shewanella* and *Ferrimonas* groups in Gammaproteobacteria have partially-overlapping *selB* and *selC* genes. The *selB* gene includes the complementary sequence of the tRNA^Sec^.

It was speculated that the *fdoGHI*-*selABCD* gene cluster has been transferred among the Alphaproteobacteria, Betaproteobacteria, and Gammaproteobacteria clades via horizontal gene transfer [7]. Some Gammaproteobacteria species belonging to the Rhodanobacteraceae family in the Xanthomonadales order have a SelB species resembling Rhizobiales SelBs. Thus, it was assessed whether the *selB-selC* overlapping trait was also transferred from Alphaproteobacteria to Gammaproteobacteria. Interestingly, a few groups of *Dyella* and *Luteibacter* have a *selB* gene whose stop codon lies in the middle of the overlapping tRNA^Sec^ sequence at the same position (from nucleotide residue 46 to 48) (Figure 5A). In contrast, their closely related strains have a new stop codon for the *selB* gene before the tRNA^Sec^ sequence. Thus, the *selB-selC* overlapping trait may have been conserved for a period in the two Gammaproteobacteria lineages. A similar case was found in a groundwater lineage of Nitrospirae (Figure 2), although the SelB and SelC sequences differ significantly from those of *Dyella* and *Luteibacter* as well as Alphaproteobacteria. On the other hand, the overlapping traits of *selB* with ORF1 or ORF2 were not found outside the Alphaproteobacteria class. Rather, the distribution of the ORF1 and ORF2 genes is limited to Alphaproteobacteria.

### 2.4. Reversed tRNA^Sec^ Overlapping with the selB Gene in Gammaproteobacteria

I found that the SelB sequences of *Shewanella* and a few *Ferrimonas* species have a C-terminal extension (Figure 2). Interestingly, it was revealed that the C-terminal extension results from the translation of the complementary sequence the tRNA^Sec^ sequence into amino acids (Figure 5B). The *selC* promoter (probably TTGATTcaggtttacacattttcTACTATC for *Shewanella oneidensis* MR-1) may exist around the stop codon of the *selB* gene (underlined). It is likely that the transcripts of the *selB* gene and the *selC* gene might function as the cis-encoded antisense RNAs to each other [15]. In contrast, *Photobacterium damselae*, another Gammaproteobacteria species, has a very similar *selB-selC* locus separated by a stop codon. The tRNA^Sec^ sequences of these Gammaproteobacteria resemble alphaproteobacterial tRNA^Sec^ sequences, indicating horizontal gene transfer.

### 2.5. Alignment Analysis of the selB C-Terminal Amino Acid Extensions

**Figure 6:**
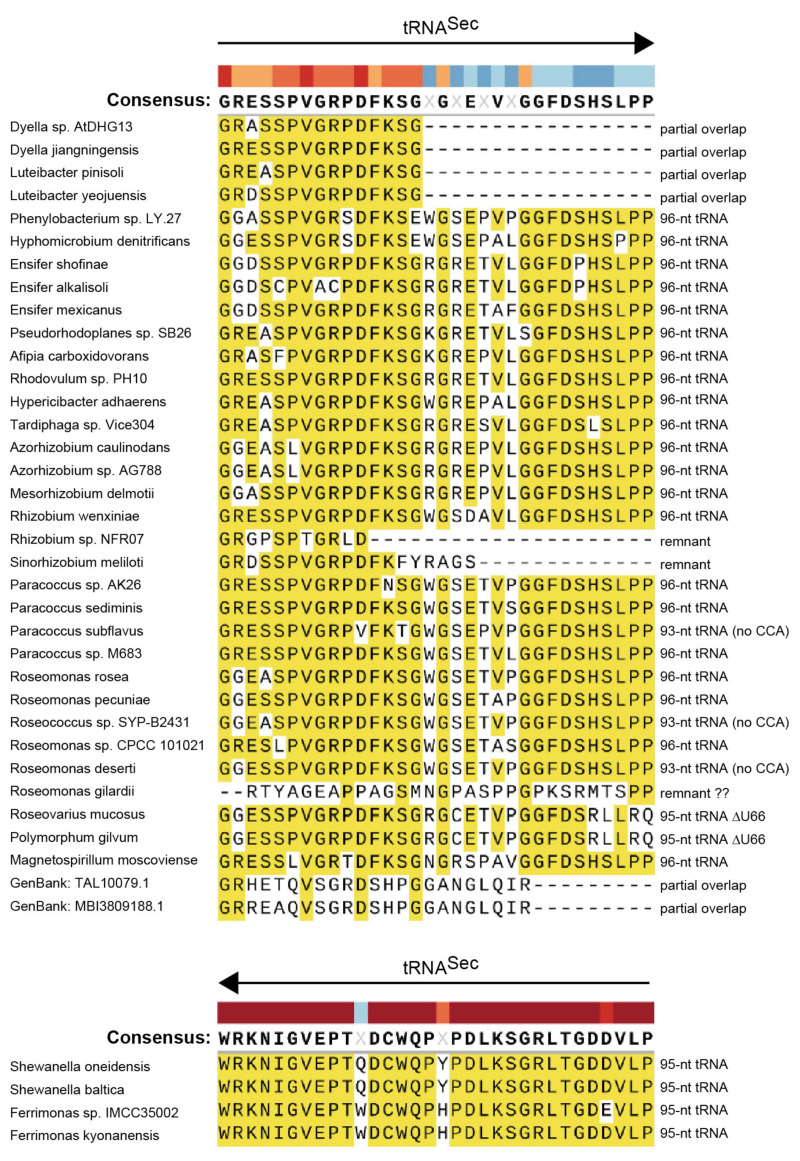
Alignment of the C-terminal amino acid extensions of SelB proteins within the tRNA^Sec^ region. Some important features of the tRNA^Sec^ sequences such as their direction, length, and completeness (with or without the encoded CCA-tail) are indicated. The 95-nt tRNA^Sec^ species of *P. gilvum* and *R. mucosus* have a non-canonical U66 deletion.

Figure 6 shows that the amino acid sequences of the C-terminal extensions of SelBs are highly conserved within the tRNA^Sec^ region. In the forward tRNA^Sec^ regions, the extensions start with Gly by translating the first three nucleotides “GGA” and end with Pro by translating the CCA tail or a remnant CCA tail sequence “CCN”. Although the number of the encoded tRNA residues is a multiple of 3 in most cases, 95-nt tRNAs were also found (Figure 6). In the partially overlapping tRNA^Sec^ regions, the positions of the stop codon for the *selB* gene are highly conserved in the *Dyella*/*Luteibacter* species and in the Nitrospirae species (Figure 6). On the other hand, in the reverse complement tRNA^Sec^ regions, the extensions start with Trp by translating the complementary sequence of the CCA end “UGG” and end with Pro by translating a nucleotide triplet “CCN” that is the complementary sequence of the nucleotides at positions from −1 to +2 of the tRNA moiety.

### 2.6. A New Mechanism of Maintaining Homeostasis between selB and Sec-tRNA^Sec^?

**Figure 7:**
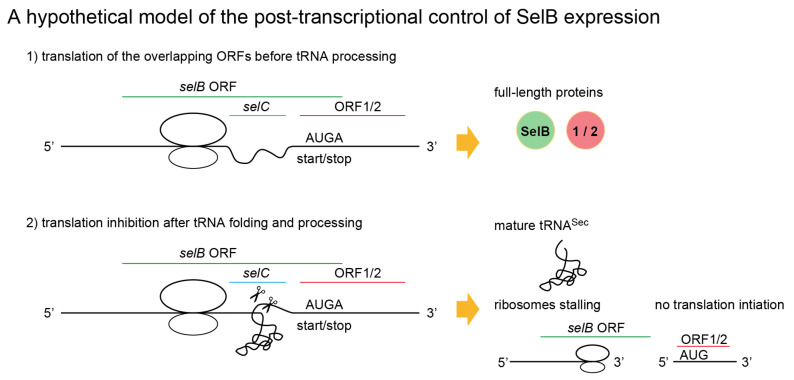
A proposed model of translational regulation of the overlapping and downstream ORFs by the nested tRNA^Sec^ moiety. The molecular ratio of tRNA^Sec^ and SelB in the cells might be kept relatively constant by the proposed mechanism.

Figure 7 shows a proposed model of the translation regulation by the nested tRNA moiety. Once the tRNA moiety folded into a tertiary structure, the exact 5′ end and the 3′ trailer may be cleaved by RNases [16,17,18]. This tRNA processing will generate a nonstop mRNA for SelB and a leader-less mRNA for ORF1 or ORF2, leading to the repression of the expression of SelB and ORF1/2 proteins (Figure 7). In other words, the expressions of SelB and tRNA^Sec^ molecules are alternatives. As discussed later, a similar mechanism was hypothesized for a mitochondrial tRNA gene nested in a protein gene [17]. Because the excess of SelB over Sec-tRNA^Sec^ may lead to sequestration of Sec-tRNA^Sec^ molecules due to the extraordinary high affinity [19,20], the SelB expression level should be maintained to be low but enough for mediating the UGA-recoding. It is not clear whether or how this hypothetical mechanism would be regulated under selenium deficiency.

## 3. Discussion

To the best of my knowledge, this is the first report of an entire tRNA sequence nested in the protein coding region of mRNA in bacteria, while tRNA-like structures of varying sizes and shapes have been found in the coding regions or untranslated regions of mRNAs in diverse organisms and viruses [21,22,23]. It is known that tRNA genes are partially or fully integrated within protein genes or other tRNA genes in the compact mitochondrial genome of animals [17,24]. For example, the 60-nt tRNA^Lys^ is fully integrated within the coding region of the cox1 gene in direct orientation in the *Armadillidium vulgare* mitochondrial genome [17]. Since Rhizobiales bacteria have a large and redundant genome, it is unlikely that they have been pressured to evolve a compact system. Rather, they may have survived selenium deficiency in the rhizosphere or inside the host plants. Plants lack the eukaryotic Sec-inserting machinery. It is also known that the gut symbionts of higher termites feeding on dead plant material often lack the Sec-insertion machinery [25] or have a putative backup system for the Sec-insertion machinery [6]. It is noteworthy that in a lineage of such symbiotic bacteria, the tRNA^Sec^ sequence ends at the -2 position of the start codon of the *selA* in the *selCAB* operon (3300006045.a:Ga0082212_10006574) [6]. Thus, expression level control of the SelA and SelB might be important in these symbiotic bacteria. The alternative expression of SelB and tRNA^Sec^ can be deemed as a new approach different from the SelB autoregulation system of *Escherichia coli* [26] for controlling the SelB expression level in bacteria. Future studies with wet-lab experiments may elucidate these mechanisms by altering the sequences such that the protein sequence would be changed without affecting the tRNA function.

## 4. Materials and Methods

The web-based BLAST tools of NCBI and the Integrated Microbial Genomes & Microbiomes system (IMG/M: https://img.jgi.doe.gov/m/) (the last accessed date: 22 April 2021) [27] were used for the bioinformatic analyses. All sequences were manually curated. The multiple alignment analysis of SelB sequences was performed using Clustal X 2.1 [28], manually curated using Seaview 4.6.5 [29], and depicted using SnapGene 5.2.4 (GSL Biotech LLC, Chicago, IL, USA). The SelB phylogenetic unrooted tree was developed by maximum likelihood estimation with 100 replicates using MEGA-X [30] using the JTT matrix-based model (bootstrap method, uniform rates, use all sites). The phylogenetic tree was drawn using FigTree v1.4.3. PyMOL 2.3.0 Open-Source (Schrödinger, LLC, New York, NY, USA) was used for the SelB rendering.

## References

[1] Labunskyy V.M., Hatfield D.L., Gladyshev V.N. (2014). Selenoproteins: Molecular pathways and physiological roles. Physiol. Rev..

[2] Silva I.R., Serrão V.H., Manzine L.R., Faim L.M., da Silva M.T., Makki R., Saidemberg D.M., Cornélio M.L., Palma M.S., Thiemann O.H. (2015). Formation of a Ternary Complex for Selenocysteine Biosynthesis in Bacteria. J. Biol. Chem..

[3] Fischer N., Neumann P., Bock L.V., Maracci C., Wang Z., Paleskava A., Konevega A.L., Schröder G.F., Grubmüller H., Ficner R. (2016). The pathway to GTPase activation of elongation factor SelB on the ribosome. Nature.

[4] Itoh Y., Sekine S.-I., Yokoyama S. (2015). Crystal structure of the full-length bacterial selenocysteine-specific elongation factor SelB. Nucleic Acids Res..

[5] Mansell J.B., Guévremont D., Poole E.S., Tate W.P. (2001). A dynamic competition between release factor 2 and the tRNA^Sec^ decoding UGA at the recoding site of *Escherichia coli* formate dehydrogenase H. EMBO J..

[6] Mukai T., Englert M., Tripp H.J., Miller C., Ivanova N.N., Rubin E.M., Kyrpides N.C., Söll D. (2016). Facile Recoding of Selenocysteine in Nature. Angew. Chem. Int. Ed. Engl..

[7] Zhang Y., Romero H., Salinas G., Gladyshev V.N. (2006). Dynamic evolution of selenocysteine utilization in bacteria: A balance between selenoprotein loss and evolution of selenocysteine from redox active cysteine residues. Genome Biol..

[8] Manian S.S., Gumbleton R., O’Gara F. (1982). The role of formate metabolism in nitrogen fixation in *Rhizobium* spp.. Arch. Microbiol..

[9] Pickering B.S., Oresnik I.J. (2008). Formate-Dependent Autotrophic Growth in *Sinorhizobium meliloti*. J. Bacteriol..

[10] Barnett M.J., Fisher R.F., Jones T., Komp C., Abola A.P., Barloy-Hubler F., Bowser L., Capela D., Galibert F., Gouzy J. (2001). Nucleotide sequence and predicted functions of the entire *Sinorhizobium meliloti* pSymA megaplasmid. Proc. Natl. Acad. Sci. USA.

[11] Copeland P.R. (2005). Making sense of nonsense: The evolution of selenocysteine usage in proteins. Genome Biol..

[12] Vargas-Rodriguez O., Englert M., Merkuryev A., Mukai T., Söll D. (2018). Recoding of the selenocysteine UGA codon by cysteine in the presence of a non-canonical tRNA^Cys^ and elongation factor SelB. RNA Biol..

[13] Selmer M., Su X.D. (2002). Crystal structure of an mRNA-binding fragment of *Moorella thermoacetica* elongation factor SelB. EMBO J..

[14] Gulevich A.Y., Skorokhodova A.Y., Ermishev V.Y., Krylov A.A., Minaeva N.I., Polonskaya Z.M., Zimenkov D.V., Biryukova I.V., Mashko S.V. (2009). A new method for the construction of translationally coupled operons in a bacterial chromosome. Mol. Biol..

[15] Bloch S., Węgrzyn A., Węgrzyn G., Nejman-Faleńczyk B. (2017). Small and Smaller—sRNAs and MicroRNAs in the Regulation of Toxin Gene Expression in Prokaryotic Cells: A Mini-Review. Toxins.

[16] Nakanishi K., Nureki O. (2005). Recent progress of structural biology of tRNA processing and modification. Mol. Cells.

[17] Doublet V., Ubrig E., Alioua A., Bouchon D., Marcadé I., Maréchal-Drouard L. (2015). Large gene overlaps and tRNA processing in the compact mitochondrial genome of the crustacean *Armadillidium vulgare*. RNA Biol..

[18] Yuan Y., Hwang E.S., Altman S. (1992). Targeted cleavage of mRNA by human RNase P. Proc. Natl. Acad. Sci. USA.

[19] Paleskava A., Konevega A.L., Rodnina M.V. (2010). Thermodynamic and kinetic framework of selenocysteyl-tRNA^Sec^ recognition by elongation factor SelB. J. Biol. Chem..

[20] Xu Y.-W., Jiang Z.-H., Mu Y., Zhang L., Zhao S.-Q., Liu S.-j., Wang C., Zhao Y., Lü S.-W., Yan G.-L. (2013). Effects of combinatorial expression of *selA*, *selB* and *selC* genes on the efficiency of selenocysteine incorporation in *Escherichia coli*. Chem. Res. Chinese Univ..

[21] Levi O., Garin S., Arava Y. (2020). RNA mimicry in post-transcriptional regulation by aminoacyl tRNA synthetases. WIREs RNA.

[22] Mukai T., Vargas-Rodriguez O., Englert M., Tripp H.J., Ivanova N.N., Rubin E.M., Kyrpides N.C., Söll D. (2016). Transfer RNAs with novel cloverleaf structures. Nucl. Acids Res..

[23] Brosius J. (1999). Transmutation of tRNA over time. Nat. Genet..

[24] Suzuki T., Yashiro Y., Kikuchi I., Ishigami Y., Saito H., Matsuzawa I., Okada S., Mito M., Iwasaki S., Ma D. (2020). Complete chemical structures of human mitochondrial tRNAs. Nat. Commun..

[25] Zhang X., Leadbetter J.R. (2012). Evidence for Cascades of Perturbation and Adaptation in the Metabolic Genes of Higher Termite Gut Symbionts. mBio.

[26] Thanbichler M., Böck A. (2002). The function of SECIS RNA in translational control of gene expression in *Escherichia coli*. EMBO J..

[27] Chen I.-M.A., Chu K., Palaniappan K., Ratner A., Huang J., Huntemann M., Hajek P., Ritter S., Varghese N., Seshadri R. (2020). The IMG/M data management and analysis system v.6.0: New tools and advanced capabilities. Nucl. Acids Res..

[28] Larkin M.A., Blackshields G., Brown N.P., Chenna R., McGettigan P.A., McWilliam H., Valentin F., Wallace I.M., Wilm A., Lopez R. (2007). Clustal W and Clustal X version 2.0. Bioinformatics.

[29] Gouy M., Guindon S., Gascuel O. (2010). SeaView version 4: A multiplatform graphical user interface for sequence alignment and phylogenetic tree building. Mol. Biol. Evol..

[30] Kumar S., Stecher G., Li M., Knyaz C., Tamura K. (2018). MEGA X: Molecular Evolutionary Genetics Analysis across Computing Platforms. Mol. Biol. Evol..

